# What outcomes of moral case deliberations are perceived important for healthcare professionals to handle moral challenges? A national cross-sectional study in paediatric oncology

**DOI:** 10.1186/s12910-022-00851-3

**Published:** 2022-11-11

**Authors:** Cecilia Bartholdson, Isabelle Billstein, Bert Molewijk, Pernilla Pergert

**Affiliations:** 1 Department of Women’s & Children’s Health, Karolinska Institutet, Stockholm, Sweden; 2 Department of Medical Humanities, Amsterdam University Medical Center, Amsterdam, the Netherlands

**Keywords:** Clinical ethics support services, Healthcare professional, Moral case deliberation, Outcome, Paediatric oncology, Survey

## Abstract

**Background:**

In paediatric oncology, healthcare professionals face moral challenges. Clinical ethics support services, such as moral case deliberation (MCD), aim to assist them in dealing with these challenges. Yet, healthcare professionals can have different expectations and goals related to clinical ethics support services.

**Methods:**

In this study, the perceptions held by healthcare professionals (nursing assistants, registered nurses, physicians, and others) regarding the importance of possible outcomes of MCDs, prior to implementation of MCDs, were investigated. A multisite, cross-sectional, quantitative study was performed at all six Paediatric Oncology Centres in Sweden. Healthcare professionals answered the Euro-MCD instrument with 26 potential MCD outcomes using a scale from Not important (1) to Very important (4). Descriptive and comparative statistical analyses were carried out.

**Results:**

All outcomes were rated high, i.e., between 3.12 and 3.78. More open communication, developing skills to analyse ethically difficult situations, better mutual understanding, and deciding on concrete actions were rated as most important. Understanding of ethical theories and critical examination of policies were rated less important. Most often nursing assistants rated higher and physicians lower than the other professions did. Women and participants without previous experience of MCDs perceived outcomes as more important. There were differences between centres as one centre had significantly higher, and one centre had significantly lower ratings compared to the others.

**Conclusion:**

It is clear that healthcare professionals want MCDs to improve teamwork and skills in order to analyse and manage ethically difficult situations. When comparing to previous research about important MCD outcomes, there were similarities in what healthcare professionals consider to be important when handling moral challenges regardless of country and potential differences in healthcare settings and systems, such as paediatric vs. adult care.

## Background

### Paediatric oncology and moral challenges

Paediatric cancer involves emotional distress, both for healthcare professionals (HCPs) and for the family [1, 2]. Paediatric cancers are life-threatening and thus, HCPs and guardians often face the moral challenge of wanting to achieve cure or to keep the child alive as long as possible, which inevitably means that the child needs to be subjected to months and often years of tough and demanding treatment. At the same time HCPs and guardians want to protect the child from unnecessary suffering. Research have shown that children in palliative care sometimes experience unnecessary suffering due to prolonged dying [3, 4]. Beside the struggling families are HCPs, who are confronted daily with complicated moral challenges that require a great mental effort to handle [5]. Moral challenges in paediatric oncology care are very broad; they are not only about life and death but are often related to balancing truth-telling, pain-management, and coercion [6]. The moral challenges often include perspectives which are different from or even conflict with those of guardians [6]. Differences and conflicts may also occur within the team regarding what the best care for the child is [7]. It is important that conflicting perspectives like these are handled in a constructive matter in the interests of good cooperation because the relationship between guardians and HCPs has been shown to affect the quality of care and health outcomes [8].

### Clinical ethics support services including moral case deliberations

Clinical Ethics Support Services (CESS) aim to assist HCPs in dealing with moral challenges in order to improve cooperation and quality of care [9]. In CESS forums difficult situations can be clarified and discussed [10], and potential outcomes of different decisions in those situations can be deliberated upon [11, 12]. One way of performing CESS is to offer Moral Case Deliberation (MCD), also referred to in the literature as Ethics Case Reflection Round [13]. MCD is an interactive meeting that serves to stimulate the shared reflections of HCPs on an ethically difficult situation and to provide a basis for sound ethical decisions [10, 12]. The ethically difficult situation often originates from cases in daily practice and gives rise to specific moral questions [14]. For example: should we continue treatment in accordance with the wishes of guardians, even though we believe the treatment to be futile? MCD has its roots in dialogical ethics and pragmatic hermeneutics [15]. Perspectives and experiences from all involved, on the specific situation, are important [10, 15], and the feedback to the moral question is based on mutual examination of the ethically difficult situation [15]. A facilitator, who can be an ethicist or a trained HCP, guides the MCD by following a structured model for ethical analysis [13, 16]. An often used structured model for ethical analysis is the Dilemma method (Table 1) [17]. The facilitator is responsible for focusing attention on the different ethical perspectives that need to be discussed and for creating an open climate where all participants can contribute to the dialogue [16, 18]. In a Dutch study, central goals of MCDs were to stimulate reflection and improve the ethical climate [19]. However, it is important to be aware of the fact that there are many different goals of CESS/MCD, which can make it difficult to implement/steer the CESS/MCD in the best way.

**Table 1:** Steps in the Dilemma method

1	Personal introduction, introduction to MCD^†^, determination of goal(s) and expectations, appointments regarding notes and confidentiality.	
2	Case presenter presents his/her case briefly [Facultative: Formulation of a general moral question].	
3	Group helps case presenter to formulate her/his dilemma: Should I do A or B? [moral burden].	
4	Participants ask questions for clarification in order to imagine what it means to be in that situation so that they can answer the dilemma question for themselves later on in the MCD^†^.	
5	Table with (non) present “perspectives/persons”, “values/motivations”, “norms/rules/actions”. Ask each participant to describe his/her values and norms with respect to the dilemma question.	
6	List all possible alternatives (without discussing feasibility).	
7	Make individual round (write down first)	a. I think the right thing to do is … b. Because … c. Therefore I’m not able to do … d. How can I cope with or decrease moral burden/damage? e. Which virtues are necessary to do the right thing?
8	Reflect upon possible group consensus or decision.	
9	Make practical appointments and plan date and place to evaluate those appointments. Distribution of the notes. Closure of MCD^†^.	
10	Evaluate in general the MCD^†^. Have we met our goals? What could be improved the next time?	

^†^ MCD: Moral Case Deliberation

By the time of data collection for this study, the joint Working Group on Ethics of the Nordic organisations in paediatric oncology for physicians/researchers (NOPHO) and registered nurses (NOBOS) had held a course in facilitating MCDs and were, in collaboration with the Paediatric Oncology Centres in Sweden, planning to implement MCDs nationwide. Until then there had not been a structured system for CESS in Swedish paediatric oncology and before implementation of MCDs it was important to investigate which specific expectations and goals HCPs had, in order to adjust the implementation and procedure of MCDs according to the results of this study.

### European MCD outcomes instrument

In 2014, Svantesson et al. developed an instrument to evaluate the quality and functionality of MCD [20]. In the European Moral Case Deliberation (Euro-MCD) Outcomes Instrument, the opinions of HCPs regarding the importance of possible outcomes of MCDs are investigated before implementation (T0). Furthermore, the Euro-MCD instrument consists of a qualitative and a quantitative section. The quantitative section (presented in this study) is completed by rating 26 possible outcomes due to MCDs. The 26 outcomes are divided in six domains: Enhanced emotional support; Enhanced collaboration; Improved reflexivity; Improved moral attitude; Impact on organizational level; and Concrete results. Each domain has three to five specific outcomes that have been selected through an extensive, iterative process consisting of a literature review, multiple Delphi rounds and validity testing [20]. Respondents are asked to answer socio-demographic questions and rate outcomes as well as listing the five most important outcomes. Response alternatives are: Very important, Quite important, Somewhat important, Not important, Cannot take a stand.

It is important to empower organizations to customize, monitor and foster implementation and quality of MCDs, that can result in fulfilling its purpose. For instance, the facilitator can adapt the MCDs according to the participants’ needs and desires, in specific contexts. Examination of possible outcomes before implementation also provides an opportunity to determine whether the opinions of HCPs regarding outcomes change after participation in MCDs.

## Method

### Aim

The aim of this study was to investigate which outcomes HCPs in paediatric oncology considered important to achieve during MCDs or subsequently in clinical practice, before having MCDs implemented. Data were investigated based on the following research questions:

- How do professions rate the 26 MCD outcomes?
- Are there differences between professions, sexes, years of experience, former experience of MCDs, and centres?

### Design

Multisite, cross-sectional survey where participants’ perceptions of MCD-outcomes were investigated using the Euro-MCD instrument [20].

### Study participants

In 2017, all six Paediatric Oncology Centres in Sweden were invited to participate in an implementation study to introduce MCDs. The centres included the university hospitals of Gothenburg, Linköping, Lund, Stockholm, Umeå and Uppsala. HCPs, n = 275, nursing assistants, registered nurses, physicians, and others (play therapist, sibling supporter, chaplain, social worker, psychologist, and managers) working at the Paediatric Oncology Centres who had regular contact with patients and families, and were likely to participate in the prospective MCDs, were invited to answer the Euro-MCD. Exclusion criteria were HCPs only working night shifts, circumstances identified by the manager indicating that an HCP was not going to be able to participate in the future MCDs or HCPs with less than one month of working experience.

### Data collection

The Swedish (T0) quantitative part of the Euro-MCD instrument [20] was used for data collection in this study. It was printed and distributed, together with an information letter, to all centres. The facilitators (registered nurses, physicians and social-workers) were about to implement MCDs at their respective centres. They assisted the research team to collect data between training sessions. HCPs were welcome to send in answers until the specific centre had introduced the MCDs. If responses were delayed, HCPs were reminded by the facilitator-trainees at their centre. The Regional Ethical Review Board in Stockholm did not see any ethical objections to the study (No: 2017/1447-31).

### Data analysis

Every questionnaire was assigned with a number based on the hospital’s city and numbers of respondents. Questionnaires with less than 10% missing answers were considered acceptable [21]. Responses rated as “Cannot take a stand” were excluded in the analyses. Statistical analyses were performed using IBM SPSS Statistics, version 25. General characteristics of the respondents and the ratings of the 26 outcomes as well as the concluding question’s “five most important” outcomes were analysed using descriptive statistics analysis (frequency, percentage, mean, SD, median, range). When analyses based on years of experience were made, the respondents were dichotomized in two ways. The first division was based on Benner’s theory of novice to expert [22] where respondents with < 5 years of experience formed one group and ≥ 5 years of experience formed the other group. The second division was based on the mean of years of experience of the group. Comparative analyses were made using non-parametric-tests with a significance level (p-value) set to < 0.05.

## Results

In total, 183 respondents completed the Euro-MCD (T0) questionnaire, which corresponded to a 67% (183/275) response rate. The characteristics of the respondents are shown in Table 2. No questionaries had more than 10% missing answers and all were therefore considered acceptable. However, two questionnaires had more than 10% responses rated as “Cannot take a stand” and were therefore excluded, resulting in the total number of 181 respondents.

**Table 2:** Sociodemographic overview of the respondents in the study

Respondents (N = 181)		n	(%)
Sex (n = 178)	Female	149	(84)
	Male	27	(15)
	Does not want to answer	2	(1)
Number of former MCD^†^ experiences (n = 170)	0	130	(77)
	1	13	(8)
	2	7	(4)
	> 2	20	(12)
Profession (n = 177)	Registered nurse	105	(59)
	Nursing assistant	45	(25)
	Physician	19	(11)
	Other	8	(5)

^†^ MCD: Moral Case Deliberation

### Characteristics of the respondents

The respondents were predominantly women, and the median age was 40 years (range 21–65). Most had not previously experienced MCDs, and regarding professional experience 18% (29/165) of the HCPs had < 5 years of experience, while 82% (136/165) had ≥ 5 years of experience (median 13, range 0–45). Regarding professions, registered nurses were the largest group, followed by nursing assistants and then physicians. Other professions included hospital play therapist, sibling supporter, chaplain, social worker, psychologist, and managers. Registered nurses were the predominant profession within both sexes, and nursing assistant was the second largest group for women, and physicians for men. The sex distribution among the professions is shown in Table 3.

**Table 3:** Number and percentage of men and women within the professional group

Profession	Registered nurse		Nursing assistant		Physician		Other	
	n = 104	(%)	n = 44	(%)	n = 19	(%)	n = 8	(%)
Women	91	(88)	39	(91)	10	(53)	6	(75)
Men	12	(12)	4	(9)	9	(47)	2	(25)
Does not want to answer	1		1					

### Perceptions of outcomes

In the whole group of respondents, all outcomes were rated as important (Table 4). The ten highest ranked outcomes were found within four domains: Enhanced collaboration, Enhanced emotional support, Improved reflexivity, and Concrete results. When comparing analyses of all outcomes with the chosen outcomes in the concluding question the results are nearly the same (Table 5).

**Table 4:** All respondents’ perceptions of the 26 outcomes descending by mean

Outcome (N = 181)	Very Important (4)	Quite Important (3)	Somewhat Important (2)	Not Important (1)		
	n (%)	n (%)	n (%)	n (%)	Median (Range)	Mean 1–4 (SD)
2. More open communication among co-workers. (n = 180)	144 (79)	33 (18)	2 (1)	1 (1)	4 (1–4)	3.78 (0.48)
1. Develops my skills to analyse EDS^†^. (n = 177)	127 (69)	48 (26)	2 (1)	0 (0)	4 (2–4)	3.71 (0.48)
8. Better mutual understanding of each other’s reasoning and acting. (n = 178)	124 (68)	48 (26)	5 (3)	1 (1)	4 (1–4)	3.66 (0.56)
13. Enables me and my co-workers to decide on concrete actions. (n = 180)	124 (68)	50 (27)	5 (3)	1 (1)	4 (1–4)	3.65 (0.56)
16. Find more course of actions. (n = 178)	115 (63)	60 (33)	3 (2)	0 (0)	4 (2–4)	3.63 (0.52)
24. Enhances mutual respect among co-workers. (n = 178)	120 (66)	47 (26)	10 (6)	1 (1)	4 (1–4)	3.61 (0.62)
9. I see the EDS^†^ from different perspectives. (n = 176)	114 (62)	50 (27)	12 (7)	0 (0)	4 (2–4)	3.58 (0.62)
4. Enables me to better manage stress caused by EDS^†^. (n = 178)	111 (61)	56 (31)	9 (5)	2 (1)	4 (1–4)	3.55 (0.65)
15. Enhances possibility to share difficult emotions and thoughts. (n = 179)	115 (63)	49 (27)	12 (7)	3 (2)	4 (1–4)	3.54 (0.70)
14. Greater opportunity for everyone to have their say. (n = 180)	103 (56)	70 (38)	6 (3)	1 (1)	4 (1–4)	3.53 (0.59)
11. Increases my awareness of the complexity of EDS^†^. (n = 179)	103 (56)	62 (34)	13 (7)	1 (1)	4 (1–4)	3.49 (0.66)
25. I become aware of my own preconceived notions. (n = 177)	102 (56)	60 (33)	14 (8)	1 (1)	4 (1–4)	3.49 (0.67)
10. I and my co-workers become more aware of recurring EDS^†^. (n = 179)	105 (57)	58 (32)	14 (8)	2 (1)	4 (1–4)	3.49 (0.69)
22. I and my co-workers manage disagreements more constructively. (n = 176)	106 (58)	53 (29)	15 (8)	2 (1)	4 (1–4)	3.49 (0.70)
23. I gain more clarity about my own responsibility in EDS^†^. (n = 179)	96 (53)	68 (37)	13 (7)	2 (1)	4 (1–4)	3.44 (0.68)
7. I feel more secure to express doubts or uncertainty regarding EDS^†^. (n = 176)	99 (54)	60 (33)	12 (7)	5 (3)	4 (1–4)	3.44 (0.75)
3. Consensus is gained among co-workers when handling EDS^†^. (n = 179)	98 (54)	63 (34)	14 (8)	4 (2)	4 (1–4)	3.42 (0.73)
18. Increases awareness of my own emotions. (n = 178)	95 (52)	65 (36)	14 (8)	4 (2)	4 (1–4)	3.41 (0.73)
26. I understand better what it means to be a good professional. (n = 174)	96 (53)	56 (31)	19 (10)	3 (2)	4 (1–4)	3.41 (0.75)
19. Strengthens my self-confidence to handle EDS^†^. (n = 177)	95 (52)	65 (36)	11 (6)	6 (3)	4 (1–4)	3.41 (0.76)
5. Contributes to the development of the workplace’s practice/policies. (n = 177)	85 (46)	78 (43)	13 (7)	1 (1)	3 (1–4)	3.40 (0.65)
20. Develops my ability to identify the core ethical question in the EDS^†^. (n = 178)	90 (49)	65 (36)	17 (9)	6 (3)	4 (1–4)	3.34 (0.79)
17. I listen more seriously to other’s opinions. (n = 177)	77 (42)	74 (40)	19 (10)	7 (4)	3 (1–4)	3.25 (0.80)
21. I and my co-workers examine existing practice/policies more critically. (n = 174)	68 (37)	82 (45)	20 (11)	4 (2)	3 (1–4)	3.23 (0.74)
6. Gives me more courage to express my ethical standpoint in EDS^†^. (n = 180)	71 (39)	80 (44)	24 (13)	5 (3)	3 (1–4)	3.21 (0.78)
12. Enhances my understanding of ethical theories. (n = 177)	62 (34)	82 (45)	26 (14)	7 (4)	3 (1–4)	3.12 (0.80)

^†^EDS: Ethically difficult situations

**Table 5:** All respondents’ “five most important” outcomes, descending by number of times chosen

Outcome (n = 174)		n‡	(%)§
13. Enables my co-workers and me to decide on concrete actions		71	(41)
2. More open communication among co-workers		65	(37)
16. Find more courses of actions		54	(31)
8. Better mutual understanding of each other’s reasoning and acting		51	(29)
1. Develops my skills to analyse EDS^†^		46	(26)
3. Consensus is gained among co-workers when handling EDS^†^		44	(25)
15. Enhances possibility to share difficult emotions and thoughts		40	(23)
4. Enables me to better manage stress caused by EDS^†^		37	(21)
19. Strengthens my self-confidence to handle EDS^†^		37	(21)
24. Enhances mutual respect among co-workers		37	(21)
10. My co-workers and I become more aware of recurring EDS^†^		33	(19)
9. I see the EDS^†^ from different perspectives		32	(18)
22. My co-workers and I manage disagreements more constructively		29	(17)
14. Greater opportunity for everyone to have their say		26	(15)
5. Contributes to the development of workplace practice/policies		25	(14)
20. Develops my ability to identify the core ethical question in the EDS^† ¶^		21	(12)
18. Increases my awareness of my own emotions		20	(12)
25. I become aware of my own preconceived notions		20	(12)
23. I gain more clarity about my own responsibility in EDS^†^		19	(11)
11. Increases my awareness of the complexity of EDS^†^		19	(11)
7. I feel more secure expressing doubts or uncertainty regarding EDS^†^		17	(10)
21. My co-workers and I examine existing practice/policies more critically ^¶^		17	(10)
26. I understand better what it means to be a good professional		16	(9)
17. I listen more seriously to the opinions of others^¶^		8	(5)
6. Gives me more courage to express my ethical standpoint in EDS^† ¶^		8	(5)
12. Enhances my understanding of ethical theories ^¶^		6	(3)

†EDS: Ethically difficult situations; ‡Number of times the outcome had been stated as one of the “five most important”; §Percentage of respondents who had stated the outcome as one of the “five most important”; ¶One of the bottom five of all 26 items (as presented in Table 4)

#### Differences between sociodemographic characteristics

Table 6 shows that compared to other characteristics the most frequently occurring differences were between women and men. Ten outcomes, representing all six domains, were rated significantly higher by women than men including three out of three possible outcomes within “Concrete results”. No significant differences were found between women and men within the group of physicians, but they did exist within the group of registered nurses (Table 7). Furthermore, in the group of nursing assistants, women rated significantly higher in one of the items: “Enables me and my co-workers to decide on concrete actions”.

**Table 6:** Significant differences between sociodemographic groups

Outcome	Mean (SD)				P-value
*Outcomes more important to women than men*	Women		Men		
1. Develops my skills to analyse EDS^†^	3.75	(0.47)	3.48	(0.51)	0.014
2. More open communication among co-workers	3.82	(0.47)	3.56	(0.51)	0.002
3. Consensus is gained among co-workers when handling EDS^†^	3.50	(0.69)	3.00	(0.85)	0.008
4. Enables me to better manage stress caused by EDS^†^	3.61	(0.61)	3.23	(0.77)	0.021
6. Gives me more courage to express my ethical standpoint in EDS^†^	3.27	(0.78)	2.89	(0.75)	0.034
8. Better mutual understanding of each other’s reasoning and acting	3.72	(0.51)	3.35	(0.75)	0.012
10. My co-workers and I become more aware of recurring EDS^†^	3.53	(0.66)	3.22	(0.80)	0.041
13. Enables my co-workers and me to decide on concrete actions	3.71	(0.54)	3.30	(0.61)	0.001
16. Find more course of actions	3.67	(0.50)	3.38	(0.57)	0.034
24. Enhances mutual respect among co-workers	3.68	(0.57)	3.23	(0.77)	0.003
*Outcomes more important according to respondents without MCD* ^‡^ *-experience (None) than those with (≥1 time)*	None		≥1 time		
5. Contributes to the development of workplace practice/policies	3.44	(0.66)	3.22	(0.62)	0.038
10. My co-workers and I become more aware of recurring EDS^†^	3.54	(0.64)	3.28	(0.75)	0.034
15. Enhances possibility of sharing difficult emotions and thoughts	3.60	(0.67)	3.33	(0.77)	0.024
17. I listen more seriously to the opinions of others	3.31	(0.76)	2.87	(0.86)	0.003
18. Increases my awareness of my own emotions	3.47	(0.71)	3.20	(0.82)	0.046
23. I gain more clarity about my own responsibility in EDS^†^	3.51	(0.65)	3.20	(0.72)	0.008
25. I become aware of my own preconceived notions	3.58	(0.58)	3.21	(0.83)	0.009
26. I understand better what it means to be a good professional	3.48	(0.68)	3.10	(0.94)	0.023

†EDS: Ethically difficult situations, ^‡^ MCD: Moral Case Deliberation

**Table 7:** Significant differences between women/men among registered nurses

Outcome	Mean (SD)				P-value
*More important according to women than men among registered nurses*	Women		Men		
1. Develops my skills in analysing EDS^†^	3.74	(0.45)	3.33	(0.49)	0.012
2. More open communication among co-workers	3.79	(0.51)	3.50	(0.52)	0.040
8. Better mutual understanding of each other’s reasoning and acting	3.69	(0.49)	3.18	(0.60)	0.010
13. Enables my co-workers and me to decide on concrete actions	3.66	(0.54)	3.25	(0.62)	0.039
18. Increases my awareness of my own emotions	3.45	(0.72)	2.91	(0.83)	0.040
19. Strengthens my self-confidence to handle EDS^†^	3.43	(0.70)	2.80	(0.92)	0.040

†EDS: Ethically difficult situations

Respondents without former MCD-experience rated eight outcomes significantly higher than respondents with former MCD-experience. Four out of the five items in “Improved moral attitude”, two of the three items in the domain “Impact on organisational level” and two of the five in the domain “Enhanced emotional support”.

Respondents with < 5 years of experience of working in healthcare rated significantly higher on four items compared to respondents with ≥ 5 years of experience. These four items were in the domains: “Enhanced moral support” and “Improved moral attitude”. When dichotomization was based on the mean of all respondents’ years of experience there was one significant difference. Respondents with longer experience rated significantly higher on one item: “My co-workers and I examine existing practice/policies more critically”.

#### Differences between nursing assistants, registered nurses and physicians

Generally, physicians rated the outcomes lower than nursing assistants and registered nurses did, except for two outcomes were physicians rated the highest including “Develops my skills to analyse ethically difficult situations” and “Mutual understanding”. In two items physicians rated in between including “Becoming aware of preconceived notions” and “Contributes to the development of workplace practice/policies”. Two of the items that differed the most were item 6: “Gives me more courage to express my ethical standpoint” where the mean for registered nurses was 3.24 and for physicians 2.76 and item 17; “I listen more seriously to the opinions of others” where the mean for nursing assistants was 3.47 and for physicians 2.89. Furthermore, nursing assistants had the highest rating in 20 of the outcomes and registered nurses had the highest ratings in 4 of the outcomes. The outcome concerning more open communication was considered to be the most important or third most important outcome among all three professions (Table 8). This outcome, together with the outcome about developing skills to analyse ethically difficult situations, occurred within all three professions’ top five highest mean outcomes. The items rated as top five among registered nurses were all present in the top five outcomes either of the nursing assistants or of the physicians (Table 8). Significant differences between nursing assistants, registered nurses and physicians are shown in Table 9.

**Table 8:** Ranked top 5 outcomes with highest mean for each profession

Outcome		Registered nurses			Nursing assistants			Physicians		
		**Mean**	**(SD)**	**Rank**	**Mean**	**(SD)**	**Rank**	**Mean**	**(SD)**	**Rank**
2.	More open communication among co-workers	3.76	(0.51)	**1**	3.82	(0.44)	**1**	3.68	(0.48)	**3**
1.	Develops my skills to analyse ethically difficult situations	3.70	(0.46)	**2**	3.66	(0.57)	**5**	3.78	(0.43)	**1**
16.	Find more course of actions	3.67	(0.49)	**3**				3.53	(0.61)	**5**
13.	Enables my co-workers and me to decide on concrete actions	3.62	(0.56)	**4**	3.76	(0.44)	**2**			
8.	Better mutual understanding of each other’s reasoning and acting	3.68	(0.52)	**5**				3.74	(0.45)	**2**
14.	Greater opportunity for everyone to have their say				3.67	(0.52)	**3**			
24.	Enhances mutual respect among co-workers				3.67	(0.67)	**4**			
25.	I become aware of my own preconceived notions							3.58	(0.69)	**4**

**Table 9:** Significant differences between NAs^†^, RNs^‡^, and physicians

Outcome	Mean (SD)				P-value
*More important according to NAs* ^*†*^ *than RNs* ^*‡*^	NAs^†^		RNs^‡^		
5. Contributes to the development of workplace practice/policies	3.57 (0.55)		3.32 (0.67)		0.037
12. Enhances my understanding of ethical theories	3.33	(0.71)	3.03	(0.82)	0.030
14. Greater opportunity for everyone to have their say	3.67	(0.52)	3.47	(0.57)	0.039
26. I understand better what it means to be a good professional	3.64	(0.57)	3.33	(0.75)	0.014
*More important according to NAs* ^*†*^ *than physicians*	NAs^†^		Physicians		
6. Gives me more courage to express my ethical standpoint in EDS^§^	3.27	(0.81)	2.79	(0.92)	0.047
15. Enhances possibility to share difficult emotions and thoughts	3.64	(0.65)	3.21	(0.86)	0.023
17. I listen more seriously to other’s opinions	3.47	(0.70)	2.89	(0.94)	0.016
24. Enhances mutual respect among co-workers	3.67	(0.67)	3.42	(0.61)	0.049
*More important according to RNs* ^*‡*^ *than physician*	RNs^‡^		Physicians		
6. Gives me more courage to express my ethical standpoint in EDS^§^	3.24	(0.70)	2.79	(0.92)	0.040
7. I feel more secure expressing doubts or uncertainty regarding EDS^§^	3.52	(0.67)	3.05	(0.97)	0.027

^†^ NA: Nursing Assistant; ^‡^ RN: Registered Nurse; ^§^ EDS: Ethically difficult situations

#### Differences between paediatric oncology centres

One of the centres (A) had significantly lower means on 17 of the 26 items. This included all items except one in the domains: “Enhanced collaboration” and “Improved moral attitude” as well as all items in the domain: “Concrete results”. Another centre (B) had significantly higher means on 13 of the 26 items. This was primarily in the domains: “Enhanced emotional support” and “Improved moral attitude” and none of the items in the domain: “Concrete results” (Table 10).

**Table 10:** Significant differences between centres reported using P-values

Domain	Outcome (N = 181)	Centre	
		**A vs. all other**	**B vs. all other**
Enhanced collaboration	8. Better mutual understanding of each other’s reasoning and acting	< 0.001	0.024
	24. Enhances mutual respect among co-workers	0.001	0.029
	14. Greater opportunity for everyone to have their say	0.038	
	22. I and my co-workers manage disagreements more constructively	0.009	
Enhanced emotional support	4. Enables me to better manage stress caused by EDS^†^		0.001
	7. I feel more secure to express doubts or uncertainty regarding EDS^†^	0.004	0.002
	18. Increases awareness of my own emotions		0.002
	19. Strengthens my self-confidence to handle EDS^†^	0.017	0.005
Improved reflexivity	1. Develops my skills to analyse EDS^†^	< 0.001	
	9. I see the EDS^†^ from different perspectives	0.003	0.003
	11. Increases my awareness of the complexity of EDS^†^		0.032
	12. Enhances my understanding of ethical theories	0.024	
Concrete results	13. Enables me and my co-workers to decide on concrete actions	0.038	
	16. Find more course of actions	0.001	
	3. Consensus is gained among co-workers when handling EDS^†^	0.002	
Impact on organizational level	10. I and my co-workers become more aware of recurring EDS^†^		0.011
	21. I and my co-workers examine existing practice/policies more critically	0.039	
Improved moral attitude	23. I gain more clarity about my own responsibility in EDS^†^	0.010	0.005
	25. I become aware of my own preconceived notions	0.035	< 0.001
	26. I understand better what it means to be a good professional	0.004	0.006
	6. Gives me more courage to express my ethical standpoint in EDS^†^	0.006	0.020

^†^EDS: Ethically difficult situations

There was a significant difference (p-value < 0.001) in the number of times HCPs at centre A (11/33) had participated in the MCDs compared to centre B (0/33).

## Discussion

In this study in paediatric oncology practice, specific quantitative outcomes that can be achieved during MCDs or, due to MCDs, were examined. In general, all outcomes were rated high. Outcomes concerning teamwork (such as open communication and mutual understanding) were rated the highest, together with outcomes concerning skills to analyse ethically difficult situations and to decide on concrete actions. There were both differences and similarities between professions. Women compared to men and respondents with no former experience of MCDs compared to those with former experience rated outcomes higher. Furthermore, one centre rated significantly higher and another centre significantly lower than the other centres.

When analysing the perceptions of all the respondents, all outcomes were considered as important. This might indicate a lack of these outcomes in current practice, or that these factors are important when handling ethical issues. It could also be argued that the high ratings were dependent on how respondents interpreted and understood the items in the Euro-MCD, as well as their perception of MCDs or their knowledge of what to expect from MCDs. In a similar study made in adult care settings in the Netherlands [23], the outcome regarding more open communication was also considered the most important. Other outcomes rated within the five highest mean in both studies were: “Enables my co-workers and me to decide on concrete actions” and “Better mutual understanding of each other’s reasoning and acting”. Thus, it seems that there are similarities in what HCPs consider to be important when handling ethical issues, regardless of country and potential differences in healthcare settings and systems, such as paediatric vs. adult care.

The results that all outcomes were rated high raises the question of to what extent MCDs can comply with these expectations. It can be presumed that there is a great opportunity in MCDs to achieve the requirement to decide on concrete action because it is one of the final steps in the Dilemma method [17], used to structure the ethical analysis during MCDs. It is also likely that the participants will develop their skills to analyse ethical issues and to see the issues from different perspectives because this is inherent in the method and the process of the MCDs; in the Dilemma method different values and norms from both MCD participants and stakeholders in the specific case at hand are made explicit [17]. Additionally, previous research [14] shows that participants in MCDs experience developing ethical competence and become more secure in handling moral difficulties, which is similar to the domain “Improved reflexivity” in this study.

Four out of five items in the domain “Enhanced collaboration” belonged to the top ten outcomes. This can indicate that HCPs see an opportunity for teamwork improvement in the sense of more open communication and enhanced mutual respect and understanding. Since communication and time for interaction are important for enhanced collaboration [24], there is a great chance of improving collaboration and teamwork through MCDs. Indeed, research has shown that MCDs do in fact improve communication and stimulate critical reflection [25], as well as improve team cooperation [26]. However, there are several challenges to performing and participating in MCDs. Previous research of potential enablers and barriers for successful MCDs, at Swedish healthcare units, showed that current hierarchal structures, present at healthcare units, contribute to difficulties in inter-professional interactions during MCDs [18]. Even if the hierarchal structures are considered to be of low degree, it might prevent the development of mutual respect by inducing negative attitudes and making it difficult for people to understand the perspective of another person, which then can hinder the outcomes that our current respondents see as important [24]. In the end this might prevent enhanced collaboration.

It has been shown that lack of motivation to interact as well as certain attitudes are barriers to collaboration [27], factors that might be present at healthcare units which cannot be influenced solely through MCDs. In order to circumvent and/or change some of these existing circumstances in MCDs, and in order to increase the chance of realizing the top ten MCD outcomes as presented in this study, the facilitator needs to create a well-functioning climate and dialogue to enable enhanced collaboration. Haan et al. [28] concluded that the most important enabling factor appeared to be the facilitator in the MCDs. For a facilitator to be able to achieve a well-functioning climate and dialogue, high demands are placed on the facilitator’s education; the education must serve to improve the facilitator’s knowledge of group dynamics and leadership. If these demands are not met, already existing tensions between professions might evolve [28] and the chances of fulfilling the important MCD outcomes decrease.

The outcome with the lowest mean was understanding of ethical theories. One potential reason for this might be that the HCPs already feel that they have enough knowledge in this area, but it might also indicate that, in the eyes of the respondents, learning about theories does not necessarily contribute to actual handling of ethical issues in practice. In the core of dialogical methods, such as MCDs, the assumption is that ethical reasoning begins with experience from practice and the clinical case, rather than theories [13, 29].

There are distinct differences in how nursing assistants and registered nurses on the one hand, and physicians on the other, rate the outcomes. This is in accordance with the study made in the Netherlands [23], where physicians also rated outcomes the lowest. The fact that nursing assistants and/or registered nurses rate all outcomes, except for two, higher than physicians in this study might indicate that they are in a greater need of a forum for handling ethical issues than physicians. Previous research [6] discussed how physicians had routines for discussing ethical issues together with their physician-colleagues more regularly than other professions, which might contribute to their somewhat smaller expectations and less positive attitude for MCDs in the multi-professional setting. This is also important because a review of the impact of MCDs on healthcare settings [28] showed that the attitudes of participants and their willingness to deliberate were important in order for MCDs to be successful.

Despite professional differences in ratings, there is an agreement among the three professions as to what outcomes of MCDs are the most important and thus should be aimed for. As we have discussed above, the Dilemma method includes a step to make practical plans for actions. If this step is performed, the outcome “Enables my co-workers and me to decide on concrete actions” is likely to be achieved. However, this step is one of the last in the method and as HCPs often are facing time pressure [18] there is a risk that there is not enough time for this step during MCDs. Also, previous research has shown that nurses want to find solutions to the issues discussed [30] and the results from the present study confirm that the timing of the MCDs needs to be planned so that there is enough time to reach this important outcome.

In the present study, women tended to rate outcomes higher than men, something also present in the Dutch investigation [23]. Thus, further analyses based on sex and profession were made. No significant differences were found within the group of physicians who were divided equally between the sexes. In the group of nursing assistants, there was a difference in one item, but this group consisted of very few men. When comparing women and men among registered nurses, multiple significant differences appeared, for example “Strengthens my self-confidence to handle ethically difficult situations” and “Develops my skills in analysing ethically difficult situations”. Thus, it might be suggested that the differences between the sexes in the whole group depended on the differences in the group of registered nurses. Additionally, the number of men among registered nurses was small, making the division of sexes within the group not evenly distributed, as with the nursing assistants, which indicates that further research is needed to make any final conclusions regarding differences associated with sex.

Nursing assistants and registered nurses rated higher than physicians on 22 of the 26 items. Items about courage in expressing one’s ethical standpoint and the possibility to share difficult emotions and thoughts, were among these. The first outcome could be due to the hierarchy which has been previously discussed. As the final medical decision always lies with the attending physician [18], it is possible that physicians express their ethical standpoint more often than nursing assistants and registered nurses. Regarding the second outcome, previous research has shown that the professions working closer to the patient often experience a greater amount of moral distress [31], thus it is likely that nursing assistants and registered nurses also experience a greater amount of difficult feelings and therefore have a greater need to deliberate emotions and thoughts than physicians. As it is often the nursing assistants and registered nurses who perform care based on the physician’s treatment decisions, they also have to deal with the patient’s reactions and the consequences of these decisions. Handling these reactions and the powerlessness of not being the decision-maker might also be a reason why they have a greater need for deliberation in this respect.

The results of this study show that there also were differences in perceptions of important outcomes between centres. The centre with lower expectations was also one of the centres where several HCPs had previous experiences of participating in MCDs. This is in line with the results of the whole group that those with previous experiences of MCDs rated lower than those without experience. This could possibly be explained by more realistic expectations and demonstrate the importance of asking for a previous MCD experience, which was noted in the Dutch study using the same instrument [23].

### Strengths and limitations

A strength with this study was that all six Paediatric Oncology Centres in Sweden participated. The response rate (67%) could be considered as quite high because research within this subject has a tendency to have low response rates [32] and previous research suggested that an average of 48.4% with a standard deviation of 20% should be used as a norm [33].

Even though the questionnaire had a short description of what MCDs implied, it is difficult to determine how easy it was for the HCPs to understand what MCD means in order to rate the outcomes before participation in MCDs. This could be further elaborated on through interviews. Furthermore, there is now a new version of the instrument (Euro-MCD 2.0) that has been modified by the original research-group. This Euro-MCD 2.0 Instrument omitted questions of perceived importance and instead focussed on the current situation regarding the outcomes, for example “We openly express our viewpoints…” [23].

A limitation of the study was that professions (for example social workers, play-therapists), other than nursing assistants, registered nurses, and physicians, only represented 4% of the total group. Hence, even though their answers could be used in the analysis of the whole group, no analysis could be performed on their answers separately. The low participation of physicians compared to nursing assistants and registered nurses was also a limitation when making statistical comparisons. However, this division, with more registered nurses and nursing assistants than physicians, is in accordance with the composition of professions in Swedish healthcare. During a day shift one unit might have 5 registered nurses, 5 nursing assistants and 2 physician working together, for example.

Differences between sexes within the professions were difficult to value because men only accounted for about 10% of registered nurses and nursing assistants. Within the group of physicians, it was equal between women and men which help to achieve to more reliable results. This finding has not been demonstrated before.

## Conclusion

MCDs aims to help HCPs deal with ethical issues and this research has demonstrated that, according to HCPs, the most important outcomes of MCDs are related to enhanced collaboration, concrete actions, reflexivity, and emotional support. Thus, managers, educators, clinical ethics support staff, facilitators and participants could focus on these outcomes to better fit with preferences of MCD participants in Swedish paediatric oncology. Interestingly, there are differences between professions and centres in terms of the average rating of outcomes. Physicians tend to rate outcomes somewhat lower, but professionals share perceptions of which outcomes are most important to achieve, the same applies for the differences between centres. The results of this investigation will be important when implementing, performing, and evaluating MCDs in paediatric oncology organizations, as well as when performing continued research using the same instrument.

## Data Availability

The datasets used and/or analysed during the current study are available from the corresponding author on reasonable request.
